# The role of APE/Ref-1 signaling pathway in hepatocellular carcinoma progression

**DOI:** 10.3892/ijo.2014.2589

**Published:** 2014-08-08

**Authors:** ZHEN YANG, SUN YANG, BOBBYE J. MISNER, FENG LIU-SMITH, FRANK L. MEYSKENS

**Affiliations:** 1 Shandong Provincial Hospital Affiliated to Shandong University, Jinan, Shandong 250021, P.R. China; 2 Chao Family Comprehensive Cancer Center, University of California-Irvine, Irvine, CA, USA; 3 Department of Pharmaceutical Sciences, University of California-Irvine, Irvine, CA, USA; 4 Department of Epidemiology, University of California-Irvine, Irvine, CA, USA; 5 School of Medicine, University of California-Irvine, Irvine, CA, USA; 6 Department of Pharmacy, Children’s Hospital of Orange County, Orange, CA, USA

**Keywords:** hepatocellular carcinoma, apurinic/apyrimidinic endonuclease-1/redox effector factor 1, copper

## Abstract

Hepatocellular carcinoma (HCC) is responsible for a third of the estimated cancer-caused deaths worldwide. To deeply understand the mechanisms controlling HCC progression is of primary importance to develop new approaches for treatment. Apurinic/apyrimidinic endonuclease-1/redox effector factor 1 (APE/Ref-1) has been uncovered elevated in various types of cancer, including HCC. Additionally, HCC progression is always correlated with elevated copper (Cu). Our previous data demonstrated that Cu treatment initiated APE/Ref-1 expression and its downstream targets. Therefore, we hypothesized that APE/Ref-1 may be involved in HCC progression through mediating the effect of Cu to its signaling cascades. Following different treatments, human HCC cell line (Hep3B) and immortalized non-malignant hepatocyte cell line (THLE3) were analyzed to explore the role of APE/Ref-1 signaling pathway. Unstained human tissue microarrays (TMA) were subjected to IHC analysis to study the relationship between APE/Ref-1 expression and clinic features. APE/Ref-1 was upregulated in HCC cells consistent with the strong expression of APE/Ref-1 in HCC tissue microarray. Greater cytoplasmic accumulation of APE/Ref-1 was found in poorly differentiated and more aggressive tumors. Also we provide evidence to show that APE/Ref-1 signaling pathway stimulates cellular proliferation, enhances antiapoptosis, and facilitates metastasis through experimental knockdown of APE/Ref-1 using siRNA in Hep3B cells or overexpressing APE/Ref-1 in THLE3 cells. These results define a novel role of APE/Ref-1 in HCC progression as being an important mediating and potentiating molecule, and also provide a basis for further investigations utilizing appropriate APE/Ref-1 inhibitors in combination with chemo-drugs for HCC treatment.

## Introduction

Hepatocellular carcinoma (HCC) is one of the most aggressive neoplasms, and is responsible for a third of the estimated cancer-caused deaths worldwide (1). Although many new chemotherapeutic agents and treatment modalities are now being used clinically, the survival rate has not improved during the past two decades. Therefore, a deeper understanding of the mechanisms controlling HCC progression is of primary importance to develop new approaches for treatment (2,3).

Apurinic apyrimidinic endonuclease/redox effector factor 1 (APE/Ref-1) is a master regulator of cellular response to oxidative stress conditions with diverse function through transactivating numerous seminal transcription factors involved in cell proliferation, apoptosis, and metastasis, such as AP-1 and nuclear factor-κB (NF-κB) (4). Therefore, the multifunctional activities of APE/Ref-1 make it a prime candidate for gene/protein therapy in clinical settings. Furthermore, emerging evidence indicates that APE/Ref-1 has been uncovered elevated in various types of cancer, and its subcellular distribution is closely correlated with tumor aggressiveness, resistance to radiotherapy, and poor outcome (5–11). However, attempts to achieve an APE/Ref-1^−/−^ human cell line or APE/Ref-1 knockout mice were embryonically lethal, suggesting that development of an APE/Ref-1 specific inhibitor instead would have significant therapeutic potential. In our study, APE/Ref-1 is not only unregulated in HCC cells but also in liver cancer tissue microarray; however, the functional role of APE/Ref-1 in tumor pathogenesis and progression is not yet clear.

In particular, it is well known that the liver is the main organ for metabolism of copper (Cu), which has long been considered to have carcinogenic potential (12). In just one example, Cu accumulation in LEC rat liver leads to spontaneous HCC (13). Conversely, Cu-depleted animals developed small, relatively avascular tumors with reduced invasive capability (14). *In vitro* studies confirmed that people with higher Cu levels are more susceptible to cancer-related mortality (15), consistent with the finding that both serum and tumor Cu levels are elevated in a variety of malignancies, including HCC (16–18). Additionally, a number of clinical trials with Cu chelators have been conducted, and the results are promising (14,19,20). Making use of this information, the present study was aimed at examining the functional role of APE/Ref-1 in HCC with or without the aid of Cu. Our data elucidate further the strong association among Cu, APE/Ref-1 and HCC, and also reinforce the hypothesis that APE1/Ref-1 is of great significance to facilitate HCC progression.

## Materials and methods

### Cell culture

Human HCC cell line (Hep3B) and immortalized non-malignant hepatocyte cell line (THLE3) were purchased from the American Type Culture Collection (ATCC, Manassas, VA, USA) and cultured as recommended by ATCC. The passage number for Hep3B used in these experiments was ≤8, and for THLE3 was 3–5. Cells were treated at 70% confluence with fresh medium and reagents added simultaneously.

### DNA and reporter constructs

The p3xFlag-Ref-1 is a gift from Dr Kaikobad Irani (University of Pittsburgh Medical Center, Pittsburgh, PA, USA). The Ref-1 promoter reporter plasmid was constructed by ligating the pGL3-basic vector (digested with *Nhe*I and *Hin*dIII) and an *Avr*II-*Hin*dIII fragment from Ref-1 promoter plasmid pCB2 (21). The resulting plasmid is termed pRef-1-Luc.

### Reagents and antibodies

The following primary antibodies were used for western blot analysis and immunohistochemistry: APE/Ref-1 antibody was from Novus Biologicals (Littleton, CO, USA); AP-1/c-Fos, MMP-1, Bcl-2 and Snail antibodies were from Santa Cruz Biotechnology (Santa Cruz, CA, USA); p84 antibody was from GeneTex Inc. (Irvine, CA, USA); α-tubulin antibody was from Sigma Life Sciences (St. Louis, MO, USA). The following secondary antibodies were used: horseradish peroxidase (HRP)-conjugated anti-mouse antibody and anti-rabbit antibody were from Santa Cruz Biotechnology.

### Cell protein extraction and western blot analysis

Following different treatments, cells were collected and lysed as described previously (22). By using a detergent-compatible protein assay kit (Bio-Rad, Hercules, CA, USA), the protein concentration was precisely measured three times. Equal amounts of the soluble protein were subjected to SDS-PAGE and transferred to nitrocellulose membrane (Sigma Life Sciences). Specific protein was detected by antibody followed by the chemiluminescence detection reagent (Bio-Rad). Measurement of signal intensity on X-ray film was performed using an Imaging densitometer with Multi-Analyst software (Bio-Rad). For statistical analysis, all data were expressed as fold changes of the control based on the calculation as the density values of the specific protein band/α-tubulin or p84 density values. All figures showing quantitative analysis include data from at least three independent experiments.

### Immunohistochemistry (IHC) and scoring

Unstained human tissue microarrays (TMA) containing 70 cases of HCC pathologically confirmed and 10 normal tissue samples were purchased from US Biomax Inc. (Rockville, MD, USA). Antigen retrieval was performed by treatment with xylene and graded alcohols. After quenching the activity of endogenous peroxidase and blocking with 5% serum, TMA were first incubated with APE/Ref-1 as the primary antibody and then secondary antibody. Envision and DAB kits (Santa Cruz Biotechnology) were used for visualization and assessments. All IHC staining was carried out in duplicate. Nuclear and cytoplasmic staining were quantified and recorded. Three experienced postdoctoral observers scored in a blinded manner using a scoring system to measure staining (0, none; 1, faint; 2, moderate; 3, strong).

### Measurement of cytotoxicity

The cytotoxicity of Cu and DSF against THLE3 and Hep3B cells was measured using the CellTiter-Glo luminescent cell viability assay (Promega, Madison, WI, USA), based on ATP bioluminescence as a marker of cell viability. After being exposed to the reagents for different time periods, the luminescence produced by the luciferase-catalyzed luciferin and ATP reaction was detected using a Modulus™ Microplate Luminometer (Turner BioSystems Inc., Sunnyvale, CA, USA) according to the instructions. Six independent experiments were performed for each group and data were normalized to the control.

### Dual-luciferase reporter assay

APE/Ref-1 reporter construct was transfected into THLE3 hepatocytes in triplicate, and luciferase activity was analyzed after 48 h using the Dual Luciferase Reporter Assay System (Promega) in a Modulus™ Microplate Luminometer (Turner BioSystems Inc.). A Renilla luciferase plasmid (Promega) was also co-transfected as an internal control for transfection efficiency. The mean luciferase activities, normalized by cell transfection efficiencies, were calculated relative to the activities of control cells.

### Quantitative real-time reverse transcription (RT)-polymerase chain reaction (PCR)

Total RNA was isolated by using TRIzol according to the manufacturer’s protocol (Invitrogen Inc., Carlsbad, CA, USA). RNA concentration was determined by using a spectrophotometer (Thermo Fisher Scientific Inc., Waltham, MA, USA). Equal amounts of mRNA were amplified using First-strand cDNA Synthesis for Quantitative RT-PCR kit (Marligen Biosciences Inc., Ijamsville, MD, USA). Real-time-PCR was performed with iQ SYBR Green Supermix and MYIQ5 detection system (Bio-Rad) using primer pairs: Snail, 5′-ATC CGA AGC CAC ACA CTG-3′ (forward) and 5′-CAC TGG TAC TTC TTG ACA TCT G-3′ (reverse); E-cadherin: 5′-GAG GAG AGC GGT GGT CAA AG-3′ (forward) and 5′-GTT CAG GGA GCT CAG ACT AG-3′ (reverse); α-tubulin, 5′-GCG TGA TGG TGG GCA TGG GTC AG-3′ (forward) and 5′-AGG GGG GCC TCG GTC AGC AGC AC-3′ (reverse). Real-time PCR data were analyzed using the comparative Ct method. The 2^−ΔΔCt^ showed the difference between the threshold cycles of the target and an internal reference (α-tubulin).

### Transient transfection studies

Small interfering RNA (siRNA) duplexes directed against APE/Ref-1 were purchased from Invitrogen Inc. Sequences of APE/Ref-1 siRNA were as follows: sense: 5′-GUC UGG UAC GAC UGG AGU ACC-3′, antisense: 5′-UAC UCC AGU CGU ACC AGA CCU-3′. Cells in 6-well plates were transfected at 50% confluence for siRNA and at 90% confluence for DNA plasmid using Lipofectamine 2000 (Invitrogen Inc.) according to the manufacturer’s instructions. The final concentration was 60 nM (siRNA) or 4 μg per well (plasmid). The serum-free medium was replaced with regular medium 6 h later.

### Cell proliferation assay

Proliferative capacity was measured using a CellTiter AQ cell proliferation assay kit (Promega). The absorbance of the formazan product at 490 nm was recorded in a Microplate reader (Bio-Rad) and cell proliferation index was calculated in comparison to the control cells. Six independent experiments were performed for each group.

### Invasion assay

The invasiveness was assessed by measuring the number of cells migrating through a Matrigel-coated membrane with pore size 8.0 μm (BD Biosciences, San Jose, CA, USA). According to the manufacturer’s instructions, two groups of cells (control and transfected cells) were collected and reconstituted in serum-free medium (SFM) at a final concentration of 2×10^5^ cells/ml. Medium (750 μl) (10% serum included) was added to each well and 500 μl of prepared cells was added to the upper Matrigel-coated insert (BD Biosciences). After incubation for 24 h, cells were fixed with ice-cold methanol and stained with hematoxylin. Subsequently, membranes were cut off, mounted, and visualized microscopically (Olympus Optical Co. Ltd., Shinjuku-ku, Tokyo, Japan). The invading cells on each of triplicate membranes were counted and averaged for 10 randomized fields at ×400 magnification.

### Cell apoptosis assay

Following a 48-h transfection with APE/Ref-1 siRNA, Hep3B cells were trypsinized and washed once with 1X PBS, fixed in cold 70% ethanol until use. Cells were incubated in propidium iodide (PI) staining solution in dark for 30 min: 50 μg/ml PI, 0.1% sodium citrate, 50 μg/ml RNase A, 0.03% NP-40 in 1X PBS. Ten thousand total events were counted for each sample which was analyzed for apoptosis by Annexin V apoptosis detection kit according to the manufacturer’s protocol (BD Biosciences).

### Statistical analysis

Data are presented in bar plot as mean ± SD for at least three independent experiments. Correlation between APE/Ref-1 immunostaining and tumor grade or stage was evaluated. Statistical analyses (two-sample t-test and Pearson’s correlation) were carried out using R-program. All p-values were two-sided, and tests with p<0.05 were considered statistically significant.

## Results

### APE/Ref-1 is upregulated in HCC cells

**Figure 1:**
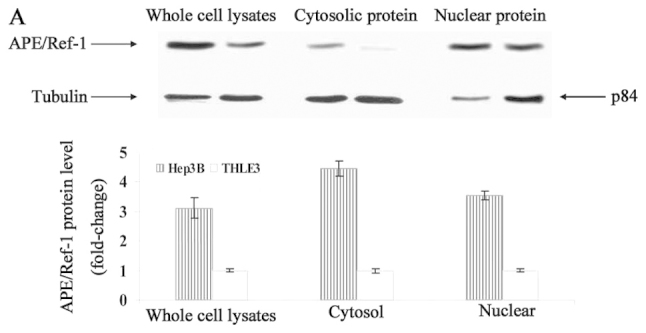

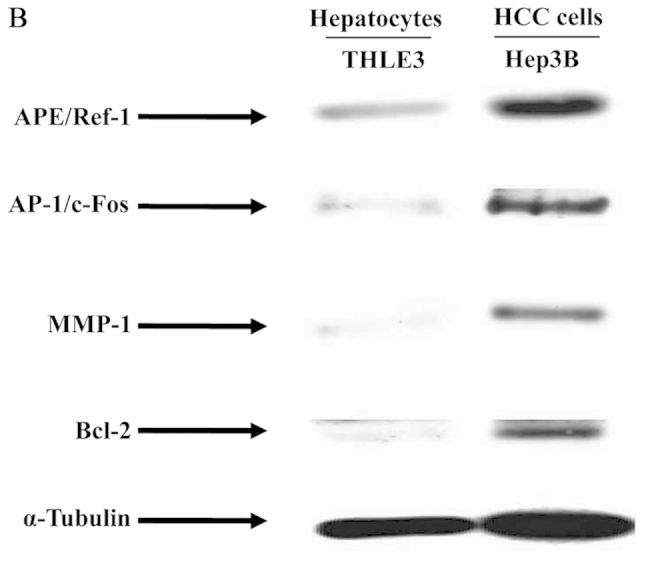
APE/Ref-1 is upregulated in HCC cells. Expression of APE/Ref-1 was analyzed using western blotting in THLE3 hepatocytes and Hep3B cells. (A) Strong expression of APE/Ref-1 in Hep3B cells assessed by western blot analysis. (B) Expression of APE/Ref-1 downstream target genes involved in cell proliferation, apoptosis and metastasis. Level in THLE3 was set at a value of 1. Data are shown as fold change of control (means ± SD). Columns, mean (n=3); bars, SD.

Expression of APE/Ref-1 was analyzed using western blotting. As shown in Fig. 1A, HCC cells exhibited a much higher level compared to hepatocytes (3.1-fold increase in whole cell lysates; 4.5-fold increase in cytoplasmic extracts; 3.5-fold increase in nuclear extracts). Furthermore, increased APE/Ref-1 level was accompanied by enhanced expression of AP-1/c-Fos, Bcl-2 and MMP-1 (Fig. 1B).

### Correlation between different patterns of APE/Ref-1 expression and clinicopathologic features

**Figure 2:**
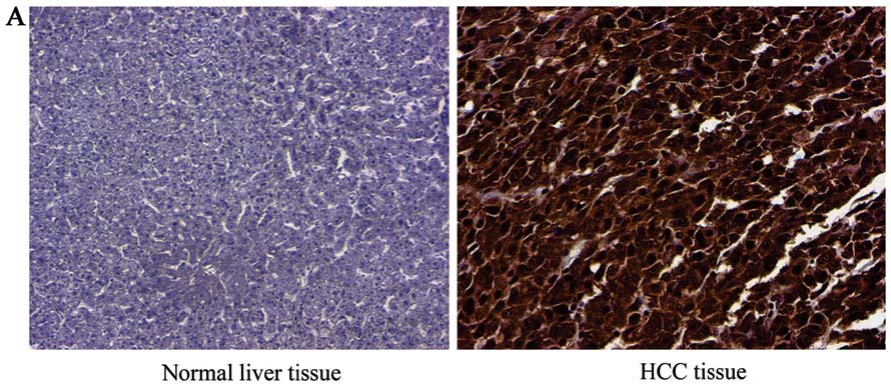

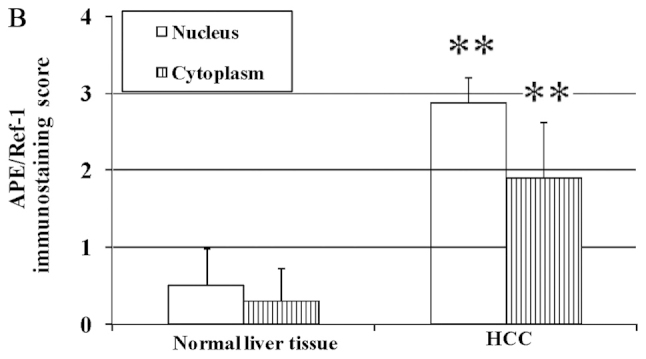

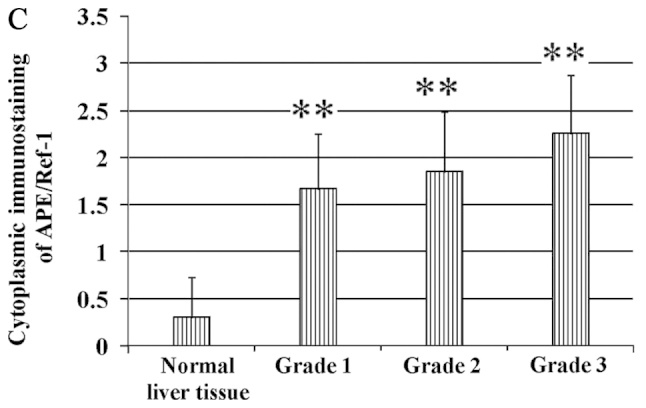

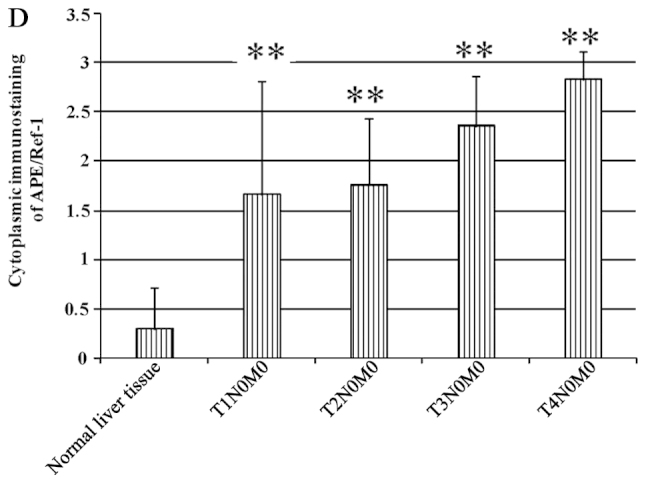
Correlation between different patterns of APE/Ref-1 expression and clinicopathologic features. Human tissue microarray was used for IHC analysis. Envision and DAB kits were used for visualization and assessments. All IHC staining was carried out in duplicate. Nuclear and cytoplasmic staining was quantified and recorded. Three experienced postdoctoral observers scored in a blinded manner at ×100 magnification based on a scoring system to measure staining (0, none; 1, faint; 2, moderate; 3, strong). (A and B) APE/Ref-1 immunostaining in normal liver tissue, and HCC tissue that exhibited a significant reactivity both in nucleus and cytoplasm (p<0.00001). Shown is a representative image with the score of +++ and +++ in the cytoplasm and nucleus (0, none; 1, faint; 2, moderate; 3, strong). (C and D) Cytoplasmic reactivity is closely correlated with tumor grades (p<0.05) and with tumor stages (p<0.001). ^*^p<0.05; ^**^p<0.01 versus control. Columns, mean (n=3); bars, SD.

TMA was subjected to IHC analysis to study the relationship between APE/Ref-1 expression and clinic features. Accumulation of nuclear and cytoplasmic APE/Ref-1 was significantly enhanced in HCC (p<0.00001, Fig. 2A and B). The average nuclear immunostaining score of APE/Ref-1 in HCC was almost 5.5-fold of normal liver tissue (NLS), and 6.3-fold for cytoplasmic staining. APE/Ref-1 reactivity was also compared in cases with different grade and stage. As shown in Fig. 2C and D, cytoplasmic staining was much higher in poorly differentiated tumors (p<0.012) and in more aggressive tumors (p<0.001); whereas no evident difference was observed in the nuclear reactivity of APE/Ref-1. These findings suggested that cytoplasmic APE/Ref-1 accumulation is closely correlated with the clinical outcome of HCC patients.

### Effects of Cu treatment on cell proliferation

**Figure 3:**
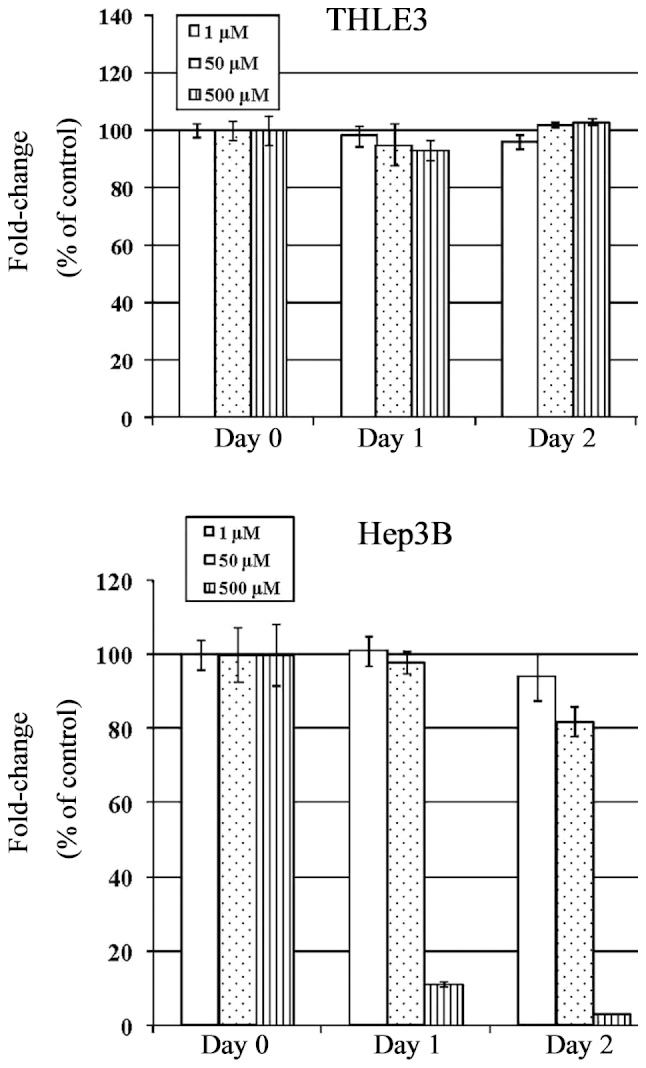
Effects of Cu treatment on cells proliferation. CellTiter-Glo Luminescent assay was used to detect the viability of hepatocytes and HCC cells after being treated for different time-points. Cu treatment initiated the proliferation of THLE3 hepatocytes even at 500 μM concentration. However, HCC Hep3B cells were much more sensitive to Cu treatment. Based on these finding, concentrations for subsequent experiments were determined as 50 μM. Columns, mean (n=3); bars, SD.

Elevated Cu levels in serum and tumors have been well documented. From our results, Cu treatment initiated the proliferation of THLE3 hepatocytes even at a 500 μM concentration. However, the HCC Hep3B cells were much more sensitive to Cu treatment, probably due to the higher Cu-load existence and consequently less buffering regulatory capacity. Based on these finding, concentrations for subsequent experiments were determined as 50 μM (Fig. 3).

### Cu treatment induces APE/Ref-1 as well as its target genes in THLE3 hepatocytes and Hep3B cells

**Figure 4:**
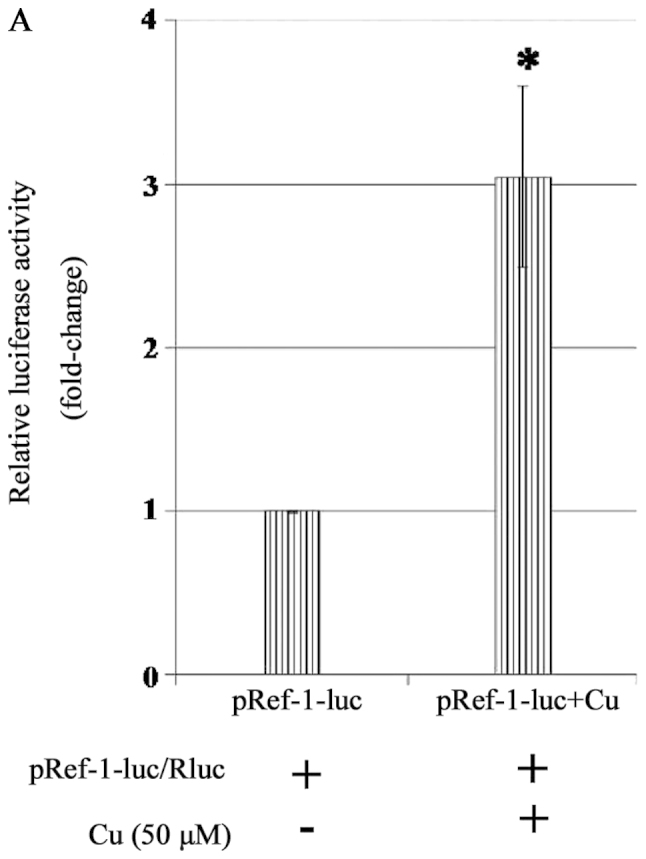

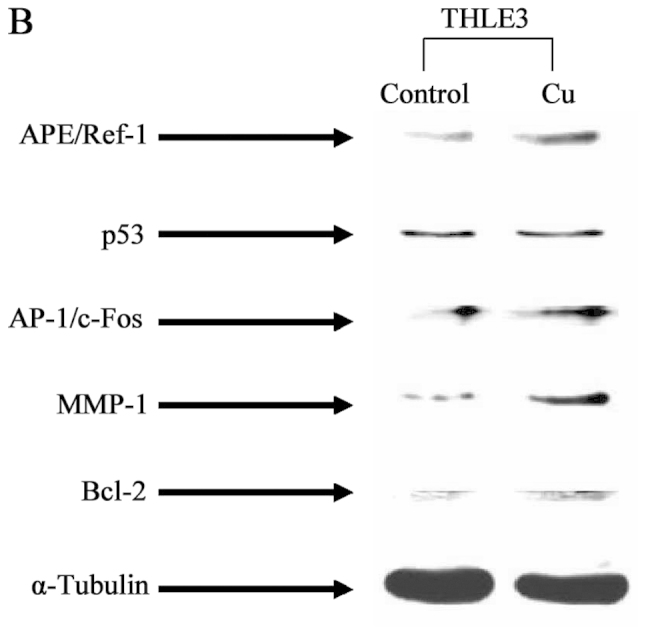

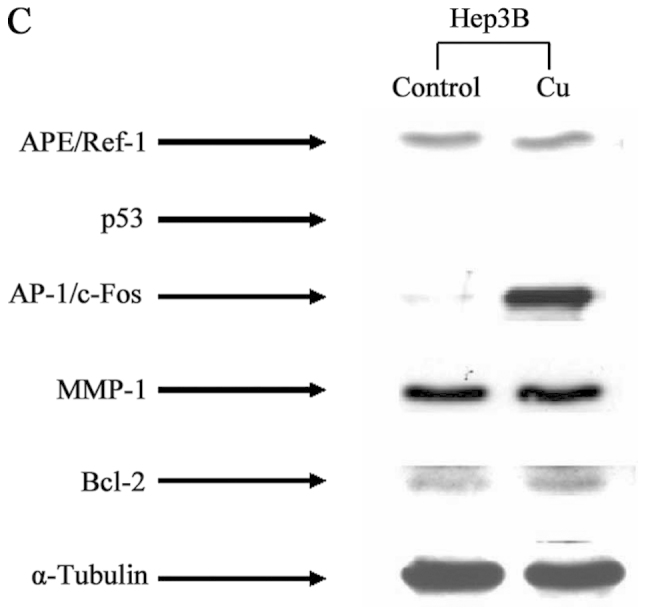

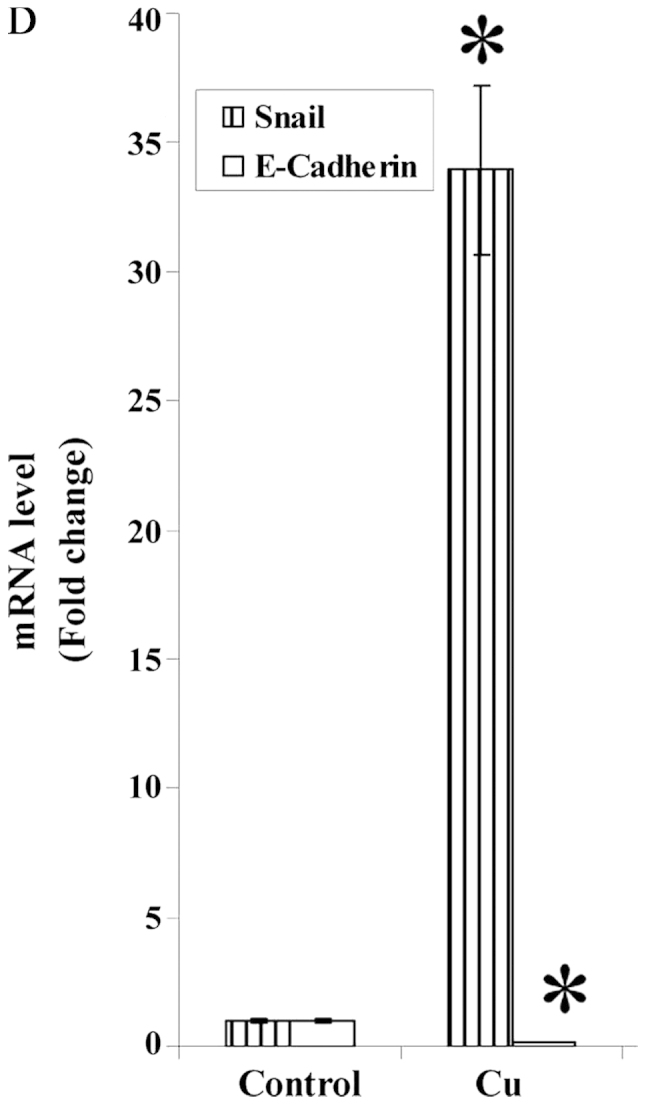

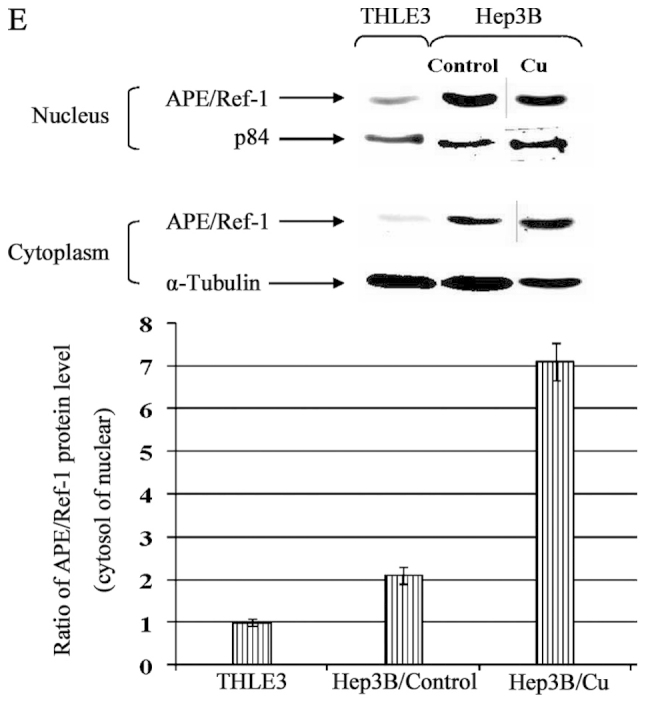
Cu treatment induced APE/Ref-1 as well as its target genes in THLE3 hepatocytes and Hep3B cells. (A) Cu potentiates promoter activity of APE/Ref-1 which was determined by Dual Luciferase Reporter assay. Induction of APE/Ref-1, AP-1/c-Fos, MMP-1 and Bcl-2 by Cu treatment in THLE3 (B) and Hep3B cells (C). (D) Quantitative real-time RT-PCR analysis of Snail and E-cadherin in Hep3B cells following 24-h incubation with Cu 50 μM. (E) Alteration of APE/Ref-1 sub-localization quantified by the ratio of cytoplasmic/nuclear APE/Ref-1 relative optical density values. Following 24-h incubation with Cu 50 μM, cells were collected and lysed. Equal amounts of the soluble protein were subject to western blotting. For statistical analysis, all data were expressed as fold changes of the control based on the calculation as the density values of the specific protein band/α-tubulin or p84 density values. Data are expressed as fold change. ^*^p<0.05; ^**^p<0.01 versus control. Columns, mean (n=3); bars, SD.

For a better understanding of the correlation between Cu and APE/Ref-1, the activity of the APE/Ref-1 promoter was measured in hepatocytes with Cu treatment using a Dual-Luciferase Reporter assay. Cu elicited an elevated luciferase activity ~3-fold that with the vector only (Fig. 4A), as well as the induction of APE/Ref-1 and its downstream signals confirmed by western blotting (Fig. 4B). In Hep3B cells, Cu treatment also induced APE/Ref-1 but much less than that of THLE3 cells (Fig. 4C). In addition, real-time RT-PCR was performed to detect E-cadherin and Snail mRNA level in Hep3B cells. Following Cu treatment, mRNA level of Snail elevated significantly along with the repression of E-cadherin (Fig. 4D). Importantly, Cu treatment led to the translocation of APE/Ref-1 quantified by the ratio of cytoplasmic/nuclear relative density values (Fig. 4E). Enhanced cytoplasmic accumulation of APE/Ref-1 was accompanied with an upregulation of MMP-1 and Snail and a downregulation of E-cadherin, proposing that Cu and APE/Ref-1 cytoplasmic distribution might be associated with HCC development.

### Targeted APE/Ref-1 depletion in HCC cells leads to decreased AP-1/c-Fos, MMP-1, Snail and Bcl-2 in parallel with corresponding alterations of proliferation, apoptosis and invasion activities

**Figure 5:**
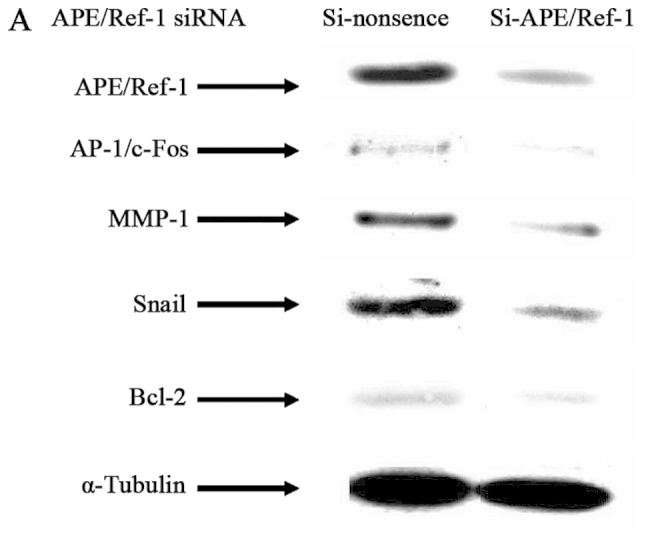

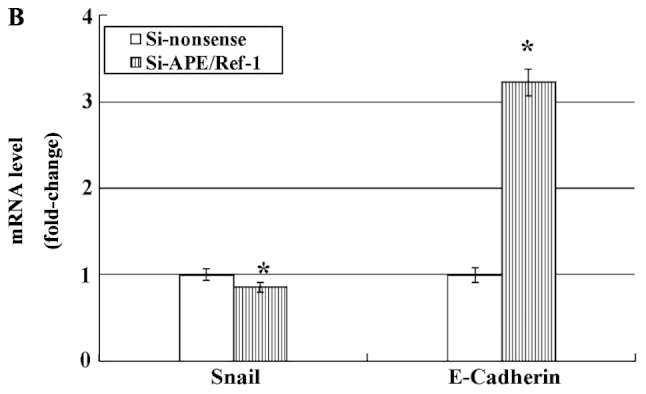

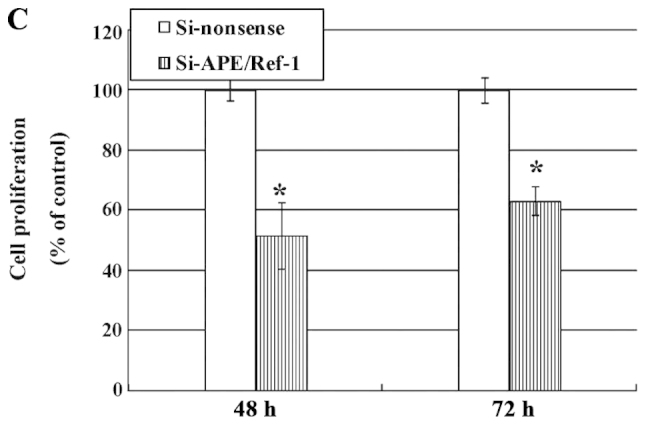

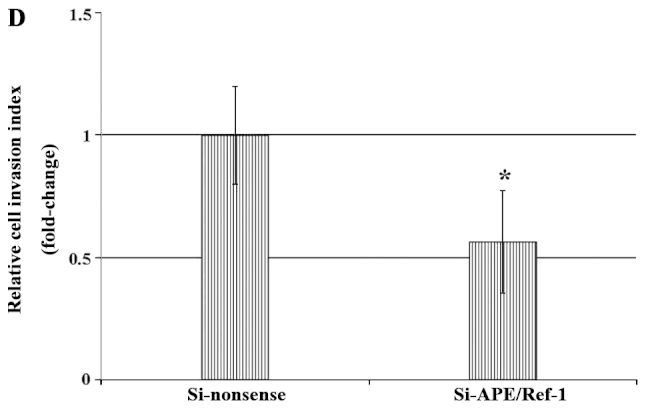

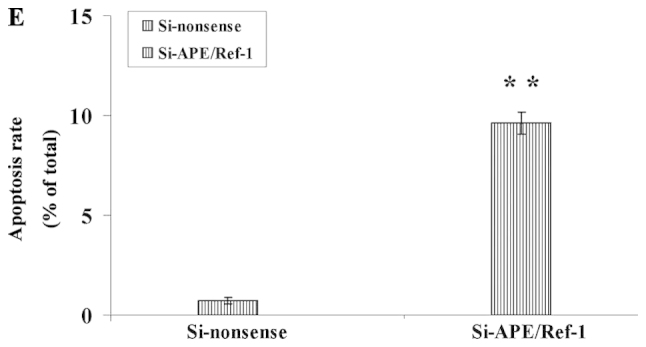
Knockdown of APE/Ref-1 by siRNA affects expression of several genes involved in its signaling cascades and inhibits cell proliferation and metastasis. (A) Immunoblots of APE/Ref-1, AP-1/c-Fos, MMP-1, Snail and Bcl-2 are shown decreased following siRNA transfection. (B) Real-time RT-PCR analysis of Snail and E-cadherin in Hep3B cells 48 h after transfection. (C and D) Targeted APE/Ref-1 depletion downregulated cell proliferation (C) and metastasis activities (D). (E) Knockdown of APE/Ref-1 induced apoptosis. Data represent the mean ± SD. ^*^p<0.05, ^**^p<0.01 versus control. Columns, mean (n=3); bars, SD.

APE/Ref-1 expression is very strong in Hep3B cells, and transient knockdown of APE/Ref-1 using siRNA was analyzed to explore the role of APE/Ref-1 in HCC. Knocking APE/Ref-1 down inhibited AP-1/c-Fos, MMP-1, Snail and Bcl-2 expression, but increased mRNA levels of E-cadherin (Fig. 5A and B). Consistently, proliferation was suppressed, invasive activity was reduced, and the apoptosis rate increased with depletion of APE/Ref-1 (Fig. 5C–E). After a 48-h transfection, cell viability decreased to 52% and invasive activity to 56% of control cells; apoptosis rate (9.6%) was 13.5-fold higher than that of control (0.71%). These data support the notion that APE/Ref-1 potentiate HCC progression by being a key mediator and regulator.

### Overexpression of APE/Ref-1 in hepatocytes potentiates the proliferative and metastatic capacities inducing its downstream targets

**Figure 6:**
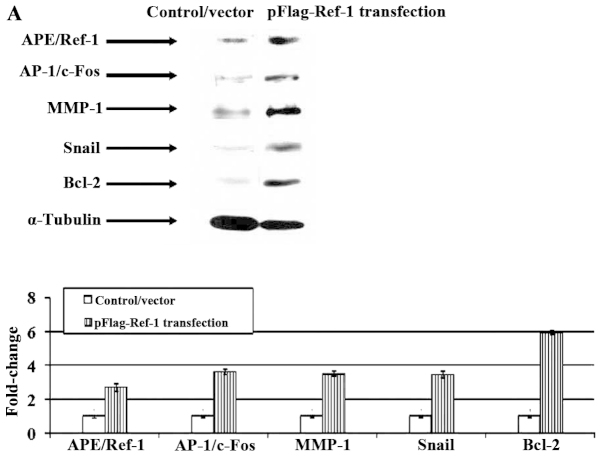

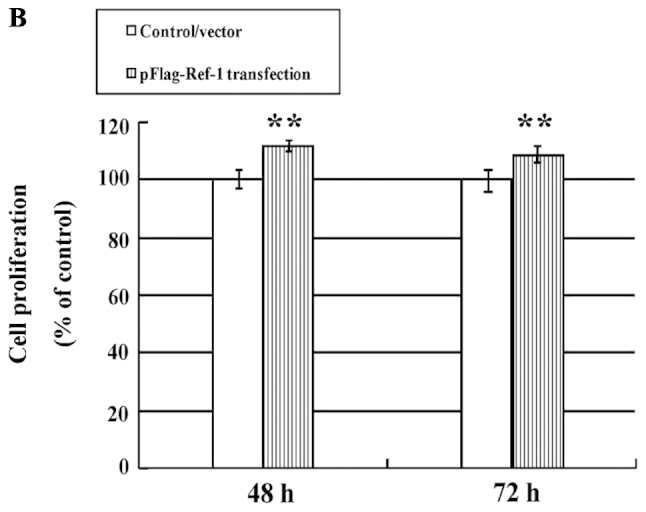

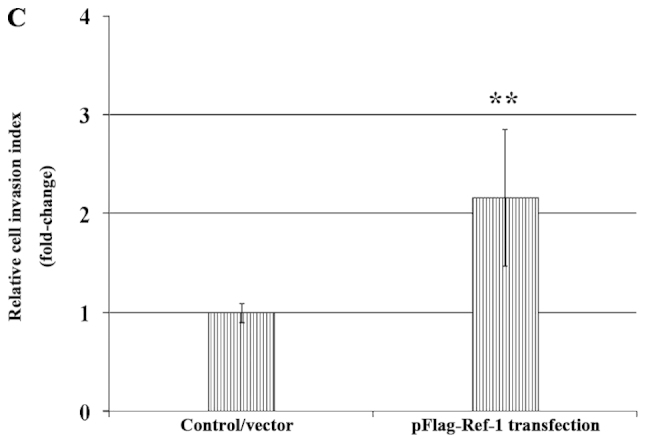
Effects of overexpressing APE/Ref-1 in hepatocytes. (A) Upregulation of APE/Ref-1 was confirmed by western blotting as well as AP-1/c-Fos, MMP-1, Snail and Bcl-2 48 h after transfection. (B) Slight influence of APE/Ref-1 overexpression on cell proliferation with a 12% increase by 48 h. (C) APE/Ref-1 positively regulated cell metastasis determined by Matrigel invasion assay. Fold change is shown as the mean ± SD. ^*^p<0.05, ^**^p<0.01 versus control. Columns, mean (n=3); bars, SD.

To further solidify the role of APE/Ref-1 in HCC progression, pFlag-Ref-1 plasmids were transfected into hepatocytes in which APE/Ref-1 expression level is very low. As shown in Fig. 6A, overexpression of APE/Ref-1 resulted in the activation of its downstream genes. There was a 3.6-(AP-1/c-Fos), 3.5-(MMP-1), 6.0-(Bcl-2), 3.5-(Snail)-fold increase. Furthermore, cell proliferation and invasiveness were assessed. By 48 h, cell viability was ~1.2-fold higher (Fig. 6B). Matrigel invasion assay confirmed that the average number of transfected cells invading the underside of the membrane was 2 times greater than the control (Fig. 6C).

## Discussion

The present study was designed to understand the role of APE/Ref-1 in HCC progression. In a Cu-rich environment, APE/Ref-1 transcription was activated, as well as the induction of its downstream signaling cascades; additionally, translocation of APE/Ref-1 from the nucleus to the cytoplasm was also observed along with the activation of Snail and repression of E-cadherin. Knocking APE/Ref-1 down reduced the expression of AP-1/c-Fos, MMP-1, Bcl-2 and Snail in parallel with corresponding decreases of cell proliferation and invasiveness, and an increased apoptosis rate. Consistently, overexpression of APE/Ref-1 in hepatocytes initiated a series of intracellular signal activations that resulted in enhanced cell proliferative and invasive capacities. Specifically, greater cytoplasmic reactivity of APE/Ref-1 was tightly correlated with a poorly differentiated (grade 3) and more aggressive tumors (stage 3–4), although both nuclear and cytoplasmic APE1/Ref-1 expressions were significantly higher in HCC tissue than in normal liver tissue. These data suggest Cu is well correlated with HCC progression in which APE/Ref-1 occupies a crucial position as an important mediator and regulator, and also presents evidence to support the prognostic role of APE/Ref-1 in the outcome of HCC patients.

It is well known that the liver is the main organ for metabolism of Cu. Excess Cu has been demonstrated to be a potent oxidizer causing production of reactive oxygen species (ROS), that in turn would create the existence of an oxidative stress related cellular disorder, such as cancer (23,24). However, the underlying mechanisms remain to be defined. In the present study, Cu promoted hepatocytes proliferation and enhanced APE/Ref-1 and its target genes expression as well, suggesting that APE/Ref-1 might be of great significance in mediating the stimulation contributing to HCC progression.

APE/Ref-1 is well documented to be very sensitive to oxidative stress and implicated in different areas, primarily as a unique link between the DNA base excision repair pathway, redox balance, transcription factor regulation and carcinogenesis (25). Aberrant enhanced expression of APE/Ref-1 appears to be a general phenomenon in human cancer (5–11). In this study, targeted depletion of APE/Ref-1 in HCC cells resulted in decreased proliferation and invasion, and increased apoptosis rate in parallel with attenuation in the expression of AP-1/c-Fos, MMP-1, Bcl-2 and Snail. On the other hand, overexpressed APE/Ref-1 in hepatocytes elicited the induction of its related signaling cascades leading cells to be more proliferative and invasive, supporting the role of APE/Ref-1 in mediating and potentiating HCC progression. Interestingly, the ratio of cytoplasmic to nuclear APE/Ref-1 accumulation changed with a marked increase following Cu treatment. In addition, our tissue array data demonstrated further that cytoplasmic intensity was closely correlated with differentiation and invasiveness, suggesting that different nuclear and cytoplasmic distribution of APE/Ref-1 should be pursued as a novel prognostic factor for clinical outcome.

As expected, AP-1/c-Fos could be regulated by APE/Ref-1; depletion of APE/Ref-1 in Hep3B cells was associated with a reduction in AP-1/c-Fos expression levels as well as cell proliferative capacity, suggesting that induced APE/Ref-1 contributes to cell proliferation through AP-1/c-Fos activation. Furthermore, depletion of APE/Ref-1 also resulted in evident attenuation of cell invasiveness, possibly mediated by decreased activities of MMP-1 and Snail. MMP-1 was considered to be closely associated with tumor aggressiveness, metastatic potential and poor prognosis (26–29). Specifically, MMP-1 expression is largely controlled at the transcriptional level through a major enhancer element consisting of a consensus AP-1 site (30–32). Therefore, it is proposed that APE/Ref-1 induces AP-1, and then MMP-1 is activated leading cells to be more aggressive and metastatic. Additionally, Hep3B cells are well differentiated and exhibited intensive MMP-1 expression, implying that MMP-1 is also needed in the early phases of invasion at the site of origin and probably plays a less critical role in the stages when such metastases are well established (33). More importantly, evidence from the literature showed that MMP-1 was induced in Snail overexpressed cells (34). Snail, the zinc-finger transcription factor, has been implicated in accelerating cancer invasion by repressing E-cadherin and increasing MMP gene expression, especially MMP-1 (34–36). In agreement with this, Snail expression was found to parallel that of MMP-1 in hepatocytes and HCC cells. Knockdown of APE/Ref-1 in HCC cells was associated with evident decreased Snail and MMP-1, and increased E-cadherin. Moreover, APE/Ref-1 overexpressing hepatocytes, Snail and MMP-1 were upregulated. Taken together, Cu exposure would induce APE/Ref-1, in turn stimulate MMP-1 and Snail, and ultimately suppress E-cadherin expression. As a result, HCC cells would be growing aggressive, leading to vascular invasion and intrahepatic metastasis (36).

In the present study, knockdown of APE/Ref-1 induced HCC cells to undergo apoptosis. We focused our attention on Bcl-2, which is an important downstream molecule regulated by NF-κB. Generally, its activation contributes to cell survival, while reduced activity promotes apoptosis in response to a variety of stimuli (37,38). In particular, it was supposed recently that apoptosis of cancer cell is mediated by p53 (39,40); however, the Hep3B cell line is p53-deficient (P53^−/−^) with stronger Bcl-2 expression, suggesting that cells depend on the upregulation of Bcl-2 mediated by APE/Ref-1 to prevent apoptosis and further facilitate survival and development.

In conclusion, one of the cellular responses to Cu stress is activation of the APE/Ref-1 pathway that regulates cellular proliferation, apoptosis and metastatic capacity. Cu exposure induced APE/Ref-1 and also changed the subcellular distribution of APE/Ref-1 protein from the nucleus to the cytoplasm. Overexpression or knockdown of APE/Ref-1 leads to corresponding alterations both in target gene expression and cell activities. Therefore, APE/Ref-1 overexpression in HCC might be an important event in the progression of HCC. Further investigations and development of APE/Ref-1 inhibitors as anticancer agents may be promising.

## Abbreviations

HCC: hepatocellular carcinoma
APE/Ref-1: apurinic/apyrimidinic endonuclease-1/redox factor-1
Cu: copper
ROS: reactive oxygen species
AP-1: activator protein-1
MMP-1: matrix metalloproteinase-1
NF-κB: nuclear factor κB
RT-PCR: reverse transcription-polymerase chain reaction
